# Tucano: Advancing neural text generation for Portuguese

**DOI:** 10.1016/j.patter.2025.101325

**Published:** 2025-07-23

**Authors:** Nicholas Kluge Corrêa, Aniket Sen, Sophia Falk, Shiza Fatimah

**Affiliations:** 1 Center for Science and Thought, University of Bonn, Konrad-Zuse-Platz 1-3, Bonn, 53227 North Rhine-Westphalia, Germany; 2 Helmholtz-Institut für Strahlen- und Kernphysik, University of Bonn, Nussallee 14-16, Bonn, 53115 North Rhine-Westphalia, Germany; 3 Bonn Sustainable AI Lab, Institute for Science and Ethics, University of Bonn, Bonner Talweg 57, Bonn, 53113 North Rhine-Westphalia, Germany; 4 Institute of Computer Science, University of Bonn, Friedrich-Hirzebruch-Allee 8, Bonn, 53115 North Rhine-Westphalia, Germany

**Keywords:** natural language processing, large language models, language modeling, low-resource languages, Portuguese, neural text generation, open source, tucano, gigaverbo

## Abstract

Natural language processing has seen substantial progress in recent years. However, current deep-learning-based language models demand extensive data and computational resources. This data-intensive paradigm has led to a divide between high-resource languages, where development is thriving, and low-resource languages, which lag behind. To address this disparity, this study introduces a new set of resources to advance neural text generation for Portuguese. Here, we document the development of GigaVerbo, a Portuguese text corpus amounting to 200 billion tokens. Using this corpus, we trained Tucano, a family of decoder-only transformer models. Our models consistently outperform comparable Portuguese and multilingual models on several benchmarks. All models, datasets, and tools developed in this work are openly available to the community to support reproducible research.

## Highlights

• Recent advances in NLP rely heavily on data and computational resources
• High-resource languages benefit, while low-resource languages face challenges
• This study introduces GigaVerbo, a 200-billion-token Portuguese text corpus
• A new series of Portuguese language models, Tucano, was developed

## The bigger picture

The rapid rise of AI language technologies is transforming how we communicate, access information, and interact with the digital world. Yet this revolution has largely favored languages with abundant digital resources, leaving many others, including Portuguese, behind. This imbalance deepens existing global inequalities and limits the reach of new technologies. Our work addresses this issue head-on by building one of the largest AI-ready Portuguese text datasets assembled and training new open models from scratch. By making these resources freely available, we aim to empower researchers, developers, and communities to build more inclusive tools. This effort is part of a broader vision: to ensure that all languages, not just English, can shape the future of AI in ways that reflect their cultures, values, and voices.

## Teaser

Recent natural language processing (NLP) advances have favored high-resource languages while leaving many others underrepresented. This imbalance challenges global AI inclusivity. Addressing it requires transparent efforts to develop datasets and models for low-resource languages. The authors present GigaVerbo, a 200-billion-token dataset for Portuguese, and Tucano, a family of natively pretrained large language models trained on GigaVerbo to support Portuguese NLP and promote linguistic diversity in AI.

## Introduction

Deep learning has dominated artificial intelligence research for nearly a decade, particularly in natural language processing (NLP).^1^^,^^2^ NLP is a prime example of the success of deep learning,^3^^,^^4^^,^^5^ where neural network approaches to machine learning have become the driving force behind many aspects of our current era of intelligent automation. Breakthroughs such as word embeddings^6^ and the transformer neural architecture^7^ have fueled advances across a range of NLP applications.

Another aspect of this developmental movement is using the self-supervised learning approach as an intermediate step to many NLP-related tasks.^8^ In essence, self-supervised learning is a training methodology for machine learning systems where we leverage the vastness of available unlabeled data at our disposition to create pretraining tasks where labeling can happen on the fly. This results in systems with useful and downstream-applicable representations tied to the domain they were trained on. This training approach has been responsible for some of the early breakthroughs of the field,^9^^,^^10^ which have now morphed into our standard training recipe for foundation models.^11^

Nonetheless, while the statement "leverage the vastness of available unlabeled data at our disposition to create pretraining tasks" can be true for languages like English or Chinese, where datasets can reach the $10^{13}$ tokens mark,^12^^,^^13^ and models are trained way past what scaling laws prescribe as compute optimal,^14^ the same cannot be said about the crushing majority of the more than 7,000 languages spoken around the world today.^15^ Hence, the prospect of training language models at the scale required to match what is done in such high-resource languages (even when compared to the state of the art from 6 years ago^16^) is a far-fetched goal for most low-resource languages.^17^^,^^18^^,^^19^

To bridge this gap, one approach outlined in the literature is the development of multilingual models, in which the self-supervised pretraining stage is conducted with various languages. Models like mBERT,^20^ mGPT,^21^ BLOOM,^22^ PolyLM,^23^ and Llama 3^24^ are examples of this approach. However, monolingual models trained on sufficiently large native corpora often outperform multilingual ones for low-resource languages, like in the case of Finnish,^25^ French,^26^ Catalan,^27^ and Portuguese.^19^

Yet, advances in developing low-resource monolingual language models, such as those for Portuguese, remain limited, small in scale, undocumented, lacking standardization, and often reliant on repurposing models trained behind closed doors, making it difficult for models to be appropriately compared and reproduced. In this work, we aim to address these challenges and build on existing studies to improve the status of generative language modeling research and development for Portuguese. In summary, we introduce two main contributions to the Portuguese NLP community.

(1) GigaVerbo, a large annotated text dataset for Portuguese language modeling.
(2) Tucano, a new low-resource, efficient, and effective open-source series of foundation models for Portuguese.

The following sections detail the methods and techniques used to develop and evaluate our model series and all related assets (e.g., annotated datasets and text quality filters) accompanying this work.

## Methods

### Literature review

**Figure 1:**
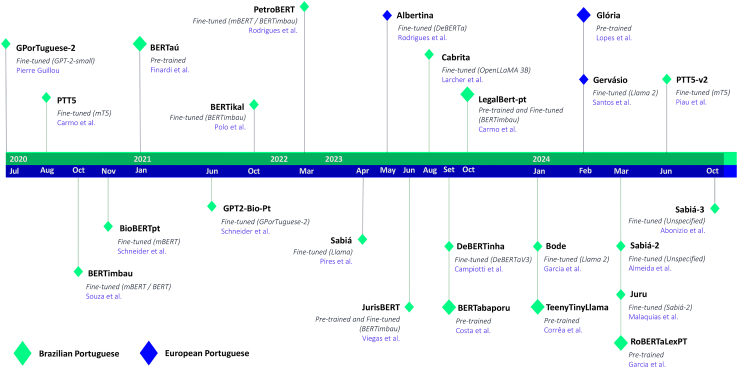
Timeline of Portuguese language model releases (2020–2024) This timeline illustrates several Portuguese language model releases from 2020 to October 2024. The models are color coded to indicate their respective Portuguese language variants, e.g., green for South America and blue for Europe. The timeline also distinguishes pretrained models from fine-tuned derivatives of other foundations. We limited the models displayed in this timeline to those we could find tied to publication reports, unpublished manuscripts, peer-reviewed papers, and popular repositories.

This subsection presents a historical overview of Portuguese large language model (LLM) research and development, providing context for our contributions. The evolution of this landscape includes numerous pretrained and fine-tuned transformer models (Figure 1).

(1) GPorTuguese-2 (July 18, 2020)^28^: the first publicly available LLM tailored for Brazilian Portuguese. GPorTuguese-2 is a byproduct of fine-tuning OpenAI’s smallest version of GPT-2^16^ on the Portuguese portion of Wikipedia. This model also has adaptations, like its own byte-pair encoding (BPE) tokenizer with a custom vocabulary that repurposes the joint embeddings from the original English vocabulary. GPorTuguese-2 was fine-tuned on ≈1.2 GB of text, and it is available under an MIT license.
(2) PTT5 (August 20, 2020)^29^: an encoder-decoder model developed as a foundation for text-to-text tasks in Brazilian Portuguese. PTT5 is an adapted version of another foundation model (Google’s multilingual T5^30^), having a custom vocabulary and embeddings that were reinitialized and trained from scratch. The PTT5 model was trained on the BrWaC corpus^31^ (≈2.68 billion tokens) and is available under an MIT license.
(3) BERTimbau (October 20, 2020)^32^: a fine-tuning version of the base and large versions of mBERT and English BERT,^20^ respectively. BERTimbau has a custom vocabulary, embeddings, and attention heads that were reinitialized and trained from scratch. Both versions of BERTimbau were trained on BrWaC^31^ and are available under an MIT license.
(4) BioBERTpt (November 19, 2020)^33^: a fine-tuned version of mBERT.^20^ BioBERTpt was created to support named-entity recognition (NER) in clinical and biomedical applications, being trained on a corpus of 44.1 M tokens of clinical narratives and biomedical-scientific papers in Brazilian Portuguese. While mBERT is licensed under an MIT license, BioBERTpt does not specify any licensing regime. However, the model is openly accessible via the Hugging Face platform.
(5) BERTaú (January 28, 2021)^34^: a pretrained BERT-based LLM for Brazilian Portuguese. BERTaú was pretrained using customer service conversations from a Brazilian financial services company (Itaú) with 5 GB of text, following a similar training protocol to the one described in the original BERT paper.^20^ As far as we could investigate, BERTaú is not open to the public, being proprietary software from Itaú.
(6) GPT2-Bio-Pt (June 1, 2021)^35^: a fine-tuned version of GPorTuguese-2^28^ trained on 48 M tokens of clinical and biomedical literature. While GPorTuguese-2 is licensed under an MIT license, GPT2-Bio-Pt does not specify any licensing regime. However, the model is accessible via the Hugging Face platform.
(7) BERTikal (October 5, 2021)^36^: a BERT model tailored for the Brazilian Portuguese legal domain. BERTikal is a fine-tuned version of BERTimbau-base.^32^ For training, the authors used 2.6 GB of text composed of legal documents from several Brazilian courts dated from 2019 to 2020. BERTikal is currently available under an MIT license.
(8) PetroBERT (March 16, 2022)^37^: a BERT-based model adapted to the oil and gas exploration domain in Portuguese. PetroBERT has two versions, each fine-tuned over a different foundation: mBERT^20^ and BERTimbau.^32^ No model is currently available for public use.
(9) Sabiá (April 16, 2023)^38^: a series of fine-tuned models that used GPT-J^39^ and Llama^40^ as a foundation. The outcomes of this fine-tuning process are Sabiá-7b, 65B (both derivatives of Llama), and Sabiá-J (using GPT-J as a base). The Sabiá series was trained on ≈7.8 billion tokens from a filtered portion of the ClueWeb 2022 dataset.^41^ Sabiá-65B and Sabiá-J are unavailable to the public, while Sabiá-7B is available under the Llama 2 license.
(10) Albertina (May 11, 2023)^42^: a family of encoder-only transformers that use DeBERTa^43^ as a foundation. Albertina models come from Brazilian and European Portuguese, having been trained on over 2.2 billion tokens of text. Currently, the Albertina series scales from 100 M to 1.5 billion parameters, and all models are available under an MIT license.
(11) JurisBERT (July 30, 2023)^44^: a series of BERT-based models developed for the Brazilian legal domain. In this series, we find models either pretrained from scratch or adapted from BERTimbau-base.^32^ We also find adapted versions from these models that were later fine-tuned to work as sentence transformers.^45^ Even though no license is tied to these models, all are available for use via the Hugging Face platform.
(12) Cabrita (August 23, 2023)^46^: a fine-tuned version of OpenLLaMA 3B^47^ with an adapted tokenizer and extended embeddings. Cabrita was trained on 7 billion tokens extracted from the Portuguese subset of the mC4 dataset.^48^ Cabrita is available under an Apache 2.0 license.
(13) BERTabaporu (September 4, 2023)^49^: two BERT models, base and large, pretrained on Brazilian Portuguese Twitter data. These models were trained on 2.9 billion tokens, following a similar training recipe as the original BERT paper.^20^ BERTabaporu is available under an MIT license.
(14) DeBERTinha (September 28, 2023)^50^: an adapted version of DeBERTaV3,^51^ fine-tuned to be performant in Brazilian Portuguese. DeBERTinha has a custom vocabulary and embeddings trained from scratch while repurposing the other weights from the original DeBERTaV3. For training, the authors used a combination of the BrWaC^31^ and Carolina^52^ datasets, which amounted to 33 GB of text (≈3.4 billion tokens). DeBERTinha is available under an MIT license.
(15) LegalBert-pt (October 12, 2023)^53^: both a pretrained BERT and a fine-tuned BERTimbau.^32^ The training dataset contained 1.5 M samples of legal texts (12 M sentences) and was used to pretrain/fine-tune both versions of LegalBert-pt. Both versions of LegalBert-pt are available under the OpenRAIL license.
(16) Bode (January 5, 2024)^54^: both a low-rank adaptation and a full fine-tuned version of Llama 2.^55^ These models were trained on a translated version of the Alpaca dataset^56^ (i.e., 52,000 instruction-following demonstrations generated by text-davinci-003). Bode is available in two sizes, 7 and 13 billion, under the Llama 2 license.
(17) TeenyTinyLlama (TTL) (January 30, 2024)^19^: a pair of language models pretrained in Brazilian Portuguese. TTL models are based on the Llama architecture,^55^ downsized to a 160- and 460-M-parameter version. These were trained on a concatenation of publicly available Portuguese datasets called Portuguese-Corpus Instruct (6.2 billion tokens). Models, datasets, and source code for training/evaluation are available under an Apache 2.0 license.
(18) Glória (February 20, 2024)^57^: a pair of language models pretrained in European Portuguese. Glória models are based on the GPTNeo architecture,^58^ scaled to 1.3 and 2.7 billion parameters. Its training dataset comprises a concatenation of European Portuguese datasets, amounting to 35.5 billion tokens. Glória’s usage is restricted to research-only purposes, subject to the ClueWeb22 Dataset license.
(19) Gervásio (February 29, 2024)^59^: a fined-tuned version of Llama 2 7B.^55^ It comes in a European and Brazilian variant, each trained on distinct datasets designed to induce instruction-following behavior. Even though Gervásio is a derivative of Llama 2, Gervásio is currently available under an MIT license.
(20) RoBERTaLexPT (March 12, 2024)^60^: a pair of encoder-only LLMs based on the RoBERTa-base implementation,^61^ tailored for general Brazilian Portuguese language modeling and applications in the legal domain. While RoBERTaCrawlPT was pretrained from scratch on the CrawlPT corpora (i.e., a deduplicated concatenation of BrWaC,^31^ Common Crawl,^62^^,^^63^ and Oscar^64^^,^^65^^,^^66^), RoBERTaLexPT was pretrained from scratch from a combination of CrawlPT and LegalPT (i.e., a concatenation of six different Brazilian Portuguese legal text corpora^42^^,^^67^^,^^68^^,^^69^). RoBERTaCrawlPT and RoBERTaLexPT are available under a Creative Commons license (CC BY 4.0).
(21) Sabiá-2 (March 14, 2024)^70^: not much information is known about Sabiá-2, and its report only brings evaluation scores of internally held benchmarking on two models of unknown sizes, referred to by the authors as "small" and "medium." Sabiá-2 is only available to the public via a commercial API (application programming interface).
(22) Juru (March 26, 2024)^71^: a fine-tuned version of Sabiá-2 small. Juru was trained on 5.88 billion tokens from academic studies and other high-quality sources tied to the Brazilian legal domain. Juru and the dataset used to train it are not available to the public.
(23) PTT5-v2 (June 16, 2024)^72^: similar to the first iteration of PTT5, PTT5-v2 is a series of fine-tuned models, up to 3 billion parameters, based on Google’s multilingual T5.^30^ PTT5-v2 was trained on approximately 524 GB of uncompressed text for 1.7 M optimization steps (115 billion tokens), following a training regime similar to the original T5 paper. Even though no license is tied to these models, all are available for use via the Hugging Face platform.
(24) Sabiá-3 (October 15, 2024): not much information is known about Sabiá-3, and its report only brings evaluation scores of internally held benchmarking on one model of unknown size. Sabiá-3 is only available to the public via a commercial API.

Reviewing past work reveals several important trends in the current state and direction of Portuguese NLP research. First, language adaptation—the repurposing of a model’s language modeling capabilities for another language—is a widely used strategy, especially when working with already high-performing multilingual models. Most of the studies listed above focus on fine-tuning or adapting pretrained models.^32^^,^^42^^,^^46^^,^^70^ This preference likely stems from challenges associated with low-resource languages, such as limited data availability, and low-resource development environments, including restricted computational capacity. For example, until 2024, almost all studies were limited to datasets with less than 10 billion tokens, with most fine-tuning models using much less than this.

Another notable trend is the recent shift from encoder-only models (such as BERT) toward decoder-only architectures. However, a significant challenge for these newer models is the lack of standardized evaluation protocols. Many studies implement their own benchmarks and metrics, making direct comparisons difficult and limiting clarity around true model performance. Additionally, most existing Portuguese benchmarks for evaluating the few-shot capabilities of generative models are either repurposed from classification tasks designed for BERT-style models^73^^,^^74^^,^^75^ or translated versions of English benchmarks.^76^ This raises questions regarding their effectiveness in evaluating generative language models. In addition, model comparisons among Portuguese language models remain very limited, as only a few available models support low-cost and accessible benchmarking for cross-study comparisons.

In terms of dataset construction, a clear trend has emerged: combining multiple text sources and removing duplicates to build larger, scalable corpora. In 2024, we see several studies implementing this approach, giving birth to some of the first large datasets (>10 billion tokens) for Portuguese language modeling.^57^^,^^60^ However, data filtering and preprocessing methods remain primarily heuristic (e.g., hash-similarity-based deduplication, HTML removal, and mojibake correction) in most studies.^57^^,^^60^ Moreover, studies that excel in high-quality text dataset curation and filtering often do not release these datasets to the Portuguese NLP community.^71^^,^^72^

It is also worth noting that several recent works have demonstrated the advantages of pretraining models from scratch over fine-tuning/adapting existing ones,^19^^,^^49^^,^^57^^,^^60^ especially in circumstances where training data are sufficient. Nonetheless, the top-performing models still tend to be fine-tuned from foundational models whose pretraining data are undisclosed.^38^^,^^70^ This lack of transparency makes it difficult to assess which factors drive performance and how much can realistically be achieved through native pretraining alone.

### Pretraining data

This section describes the procedures used in the construction of GigaVerbo.

#### Concatenating GigaVerbo

Datasets such as those developed by Lopes et al.^57^ (35.5 billion tokens) and Garcia et al.^60^ (≈90 billion tokens) are created by concatenating and filtering multiple existing corpora—either drawn from prior studies or collected via web crawls like Common Crawl and OSCAR. This mirrors the methodology used in English-focused collections such as The Pile^77^ and MassiveText,^78^ which are also collections of large text datasets from multiple sources, but with a focus on English. We applied the same methodology to create our dataset’s initial version, concatenating several portions of openly available datasets for Portuguese and deduplicating their summation with an exact hash deduplication filter. Details on every subset of GigaVerbo can be found in Table 1.

**Table 1:** Description of the different subsets comprising GigaVerbo

Subset	No. of samples	%	Description
monoHPLT-PT	58,244,012	40.09%	the clean and deduplicated Portuguese portion of the high-performance language technologies resources dataset
CrawlPT	43,846,974	30.17%	a deduplicated Portuguese corpus extracted from various web pages, concatenated from CC-100, Oscar, and BrWaC
Multilingual-C4	16,092,571	11.07%	the Brazilian Portuguese cleaned portion of the m-C4 dataset
Common Crawl	12,470,998	8.58%	a clean and deduplicated snapshot of the Common Crawl dataset (CC-MAIN-2023-23)
BlogSet-BR	4,321,181	2.97%	a collection of blog posts written in Brazilian Portuguese
Instruct-PTBR	2,962,856	2.04%	a mix of multiple instruction datasets for various tasks, machine translated (Google Translate API) from English to Brazilian Portuguese
Corpus Carolina	2,075,395	1.43%	an open corpus with varied typology in contemporary Brazilian Portuguese
UltrachatBR	1,255,091	0.86%	a Portuguese version (machine translated by the Google Translate API) of the Ultrachat dataset
Wikipedia	1,101,475	0.76%	cleaned Portuguese articles built from the Wikipedia dumps
CulturaX	999,994	0.69%	the Portuguese portion of CulturaX, a multilingual dataset with 167 languages
LegalPT	925,522	0.64%	a concatenation of publicly available legal data in Portuguese, including legislation, jurisprudence, and legal articles
Gpt4All	808,803	0.56%	a Portuguese (machine translated by the Google Translate API) version of the Gpt4All dataset
XL-Sum	64,577	<0.1%	a Portuguese (machine-translated) version of XL-Sum, a diverse dataset for abstractive summarization
Dolly 15K	28,401	<0.1%	a Portuguese (machine translated by the LibreTranslate API) version of Dolly 15K, an open-source dataset of instruction-following records generated by human annotators
CosmosQA	25,260	<0.1%	a Portuguese (machine translated by the GPT-3.5-turbo) version of the CosmosQA dataset for commonsense-based reading comprehension
ROOTS	10,740	<0.1%	the Portuguese portion of the ROOTS corpus, a dataset spanning 59 languages

Approximately 96% of GigaVerbo is comprised of native Portuguese text (i.e., monoHPLT-PT,^79^ CrawlPT,^60^ Multilingual-C4,^30^ Common Crawl,^62^^,^^63^ BlogSet-BR,^80^ Corpus Carolina,^52^ Wikipedia,^81^ CulturaX,^82^ LegalPT,^60^ Bactrian-X,^83^ XL-Sum,^84^ and ROOTS^85^), with a minority (≈4%) of English-to-Portuguese machine-translated subsets (i.e., Instruct-PTBR,^86^ UltrachatBR,^87^ Gpt4All,^88^ Dolly 15K,^89^ and CosmosQA^90^). More information can be found in its dataset card.

#### Filtering GigaVerbo

Recent research suggests that improving dataset quality can often yield greater performance gains than simply increasing dataset size or model parameters.^13^^,^^91^^,^^92^^,^^93^ However, what defines a text as "high quality" is a nontrivial question. While heuristic-based filters can help us parse samples that are, for example, too short or ill-formatted, it is hard to differentiate high-quality text (e.g., articles, poems, and tutorials) from plain text scraped from the web (e.g., product information scraped from e-commerce platforms) using only heuristic-based filters. Since human annotation is expensive and time consuming^94^ and existing learned filters are often unsuitable for Portuguese or computationally inefficient, we opted to train our own filter, following the strategy used by Gunasekar et al.^92^

**Figure 2:**
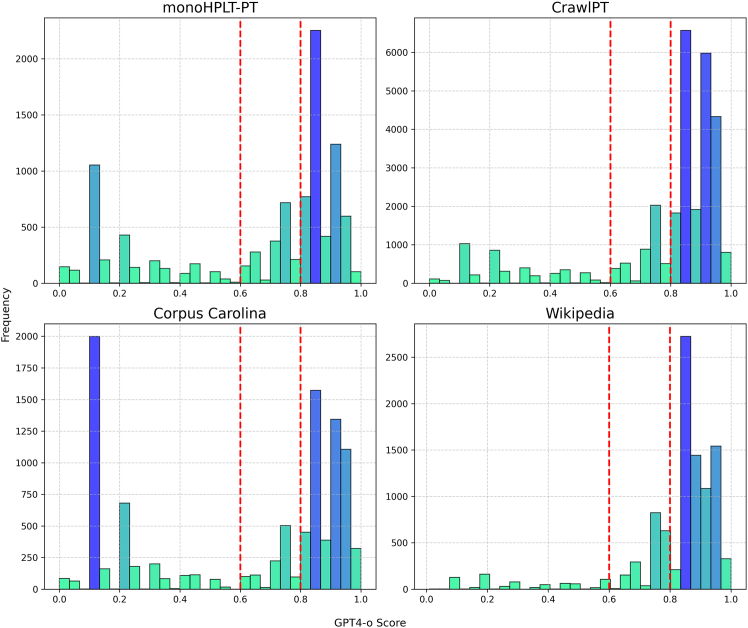
Quality assessment and toxicity filtering with GPT-4o This graph shows the distribution of scores for 4 subsets of GigaVerbo. We determined that the text would have a "high" quality if the GPT-4o scores were ≥ 0.8 and "low" when ≤ 0.6, thus keeping our dataset with a more balanced proportion of labels for our classifiers. Above, we see that datasets like monoHPLT and Corpus Carolina have some of the lowest-quality samples. Also, given that GPT-4o is extremely sensitive to toxic and harmful content, samples containing toxic, dangerous, or NSFW (not safe for work) content end up scoring very low (< 0.1), given as a way to account for the toxicity in our dataset. Analyzing samples from the Wikipedia portion scored by GPT-4o, we found that the model consistently gives low scores (<0.5) to ill-formatted, incomplete, or excessively short documents (<20 words).

For this, we randomly selected 110,000 samples from 9 subsets of GigaVerbo (i.e., specifically those not synthetic or machine translated). With these samples, we created a text-quality dataset using GPT-4o as a judge. Similar to the study of Gunasekar et al.,^92^ we prompted GPT-4o to score every text sample regarding its quality to create an annotated text-quality classification dataset for the Portuguese language (Figure 2).

As a first attempt, we sought to emulate Gunasekar et al.^92^ by converting the text samples of our classification dataset into embedding representations via a sentence-BERT. After evaluating several available multilingual sBERTs, we selected LaBSE (language-agnostic BERT sentence embedding),^95^ which generates 768-dimensional embedding vectors. Then, we trained a shallow classifier based on XGBoost. To convert real-numbered scores into labels, we binarized our data by defining as "high" all samples with a score ≥ 0.8 and "low" all those with a score ≤ 0.6. However, we were not satisfied with the results of this initial approach, and we hypothesize that the embedding representations of LaBSE were not performant enough for Portuguese. Hence, we decided to use BERTimbau^32^ as the foundation for a text classification model. The results for both approaches can be found in Table 2.

**Table 2:** Performance comparison of LaBSE + XGBoost and BERTimbau-based classifiers on GigaVerbo

Model	Class	Precision	Recall	F1-score
LaBSE + XGBoost	low	0.89	0.81	0.85
	high	0.92	0.96	0.94
BERTimbau	low	0.99	0.97	0.98
	high	0.99	0.99	0.99

The evaluation scores for both our LaBSE + XGBoost and BERTimbau-based classifiers are shown. These scores were obtained by evaluating both models on a test set of 11,000 samples. For the XGBoost, we used a learning rate of 0.1, a maximum tree depth of 10, and 100 estimators. For fine-tuning BERTimbau, we used a learning rate of 4 × 10^−5^, a weight decay of 0.01 for regularization, and a batch size of 128 for 3 epochs on our entire dataset. We also experimented with training a LaBSE + XGBoost regression algorithm, which achieved a root-mean-squared error of 0.16 on our evaluation, and fine-tuning BERTimbau-large, which achieved very similar results to its base version.

Ultimately, we chose to use our fine-tuned version of BERTimbau-base to filter GigaVerbo, given that it had achieved good performance and was faster than our XGBoost classifiers and BERTimbau-large. We applied our fine-tuned BERTimbau-base classifier to the full GigaVerbo dataset, filtering out low-quality samples. Of the 145 M samples, around 50 M were labeled as low quality, leaving approximately 65% deemed high quality. However, for this study, we adopted a filtering approach where we only removed the low-quality samples if the confidence of our classifier was above 95% for the low-quality class. We expect that this would minimize token waste due to low-confidence false negatives. This approach leaves us with ≈70% of GigaVerbo to work with. The available GigaVerbo version has the class and confidence score assigned by our filter for each text sample, allowing other users to replicate our training mixture or adapt the filtering process to their liking.

### Tokenization

As highlighted in previous studies,^19^^,^^34^^,^^46^ tokenization plays a critical role in language modeling efficiency. A tokenizer that effectively compresses text can improve both context usage and training performance. Although the exact impact on overall language modeling performance remains uncertain,^96^ tokenization undeniably plays a crucial role in this process.^97^ In terms of compression, tokenizer efficiency—measured by the number of tokens needed to encode a given text—can be significantly improved by using a vocabulary specifically tailored to a given domain.^19^^,^^46^ Optimizing for better compression helps maximize the use of limited resources, such as context length in transformer architectures.

**Figure 3:**
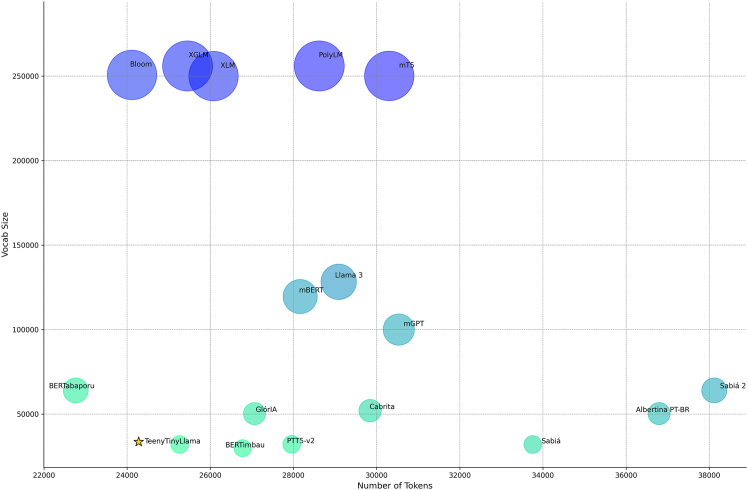
Vocabulary size and compression efficiency across tokenizers This figure lets us understand specific relationships between vocabulary size and the respective tokenizer’s capabilities regarding compression. For example, models that use the Llama 2 tokenizer (e.g., Sabiá), which is primarily focused on English, do not encode Portuguese very efficiently. On a similar note, Sabiá-2 has the worst performance across all tokenizers, even though it has doubled the vocabulary size of its predecessor. Meanwhile, multilingual models, like mBERT, PolyLM, Llama 3, mT5, and mGPT, improve their compression efficiency by having significantly enlarged vocabularies, with Bloom, XGLM, and XLM being close to the top of this comparison, all using massive multilingual vocabularies with >250,000 tokens. As a middle ground between efficiency and resource consumption (i.e., larger vocabularies imply larger embedding matrices, which then imply more computational requirements for inference or training), we have tokenizers with vocabularies tailored for the Portuguese domain (e.g., BERTabaporu, TeenyTinyLlama, and BERTimbau). In summary, while multilingual (or larger) vocabularies generally offer improved compression, small, domain-specific tokenizers balance efficiency and computational resource consumption. The code for replicating this test is available in GitHub.

To evaluate tokenizer efficiency across our curated set of Portuguese LLMs, we adopted the evaluation methodology from Larcher et al.^46^ and Corrêa et al.,^19^ applying it to various tokenizers built for Portuguese LLMs. Our evaluation used a 14,000-word sample of Portuguese poetry, including works by Fernando Pessoa, Ronald de Carvalho, and others. This choice is motivated by the rich and diverse vocabulary found in Portuguese poetry, which features unique words, complex expressions, and varied grammatical structures that highlight the depth of the Portuguese language. The results of our custom evaluation are displayed in Figure 3. This evaluation can be reproduced or adapted using the source code in our GitHub.

According to our experiments, the tokenizer trained by Corrêa et al.^19^ presents both an efficient compression capability and a slim vocabulary size for improved efficiency during input and output embedding matrices computations. The TTL tokenizer (from now on referred to as the Tucano tokenizer) is a SentencePiece tokenizer^98^ that implements both sub-word and unigram tokenization. We utilized this tokenizer to encode our pretraining dataset, separating each document with an end-of-text token (</s>) and packing the sequences up to the maximum set context length for each model.

### Architecture

Like many other studies,^19^^,^^38^^,^^46^^,^^54^^,^^70^ we used a decoder-only transformer based on the Llama architecture^24^^,^^40^^,^^55^ as the basis for our models. In terms of code implementation, we used the implementation provided by Hugging Face so that the community can easily share and use our models. Like the standard Llama architecture, our models use both root-mean-square layer normalization^99^ and RoPE (Rotary Position Embedding) embeddings,^100^ with Silu activations^101^ instead of the SwiGLU^102^ (Swish Gated Linear Unit) described in the original Llama papers. All models were trained using a causal language modeling objective and cross-entropy as their loss. The dimensions of our Tucano series are documented in Table 3.

**Table 3:** Architectural details of the Tucano series

*n*_*param*_	*n*_*layers*_	*d*_*model*_	*d*_*mlp*_	*n*_*heads*_	*n*_*KV-heads*_	*d*_*head*_	*c*_*length*_
162,417,408	12	768	3,072	12	12	64	2,048
630,253,568	14	2,048	4,096	16	4	128	2,048
1,100,048,384	22	2,048	5,632	32	4	64	2,048
2,444,618,240	24	2,560	10,240	16	4	160	4,096

Each model is based on a decoder-only Llama architecture with a vocabulary size of 32,000. Tucano-160m, -630m, and -1b1 were trained with a context window of 2,048 tokens, while the largest model (2b4) was trained with sequences of length 4,096. All models were trained using a causal language modeling objective and cross-entropy loss. The parameters were explicitly tuned to fit these models (and respective optimizers and batches) on A100-SXM4-80GB GPUs. Unlike previous studies,^19^^,^^103^ we trained all models beyond what the Chinchilla scaling laws prescribed.^104^ Group-query attention,^105^ with 4 key-value heads per decoder block, was used to reduce attention computations’ memory footprint, helping us to maximize token throughput during training without significantly impacting model convergence when training Tucano-630m, -1b1, and -2b4.

### Training hyper-parameters and performance

Our training code base was primarily built with a PyTorch-based deep learning stack.^106^ Because all model sizes fit within the available GPU memory, we adopted a straightforward distributed data-parallel (DDP) setup.^107^ For support, we used specific libraries tied to the Hugging Face ecosystem, like tokenizers^108^ for fast tokenization and datasets^109^ for handling our pretraining corpus. We also used FlashAttention^110^^,^^111^ for optimized IO (input/output) aware attention computation and the Liger Triton kernels^112^ to reduce memory footprint and improve token throughput during training. We used 8 NVIDIA A100-SXM4-80GB GPUs to train both smaller versions of Tucano (160m and 630m) and 16 of these for our two largest models (1b1 and 2b4). Lastly, we utilized CodeCarbon^113^ to track the resource consumption of our experiments and training runs.

To assess the training efficiency of our deep learning stack, we applied the MFU (model floating point operations per second utilization) methodology proposed by Chowdhery et al.,^114^ which can be understood as the ratio of the observed throughput (actual performance) to the theoretical maximum throughput that a given hardware offers. Regarding speed comparison, our code implementation is on par with other documented developments in the literature. For example, for our Tucano-1b1, we were able to achieve a training throughput of 24,180 tokens per s per A100-SXM4-80GB, which is similar to that achieved in the development of TinyLlama^14^ and superior to those documented in the development of the Pythia suite.^115^ Table 4 documents the hyper-parameter settings used to train our models and the efficiency we achieved.

**Table 4:** Training configuration and optimization metrics for the Tucano series

	160m	630m	1b1	2b4
Total optimization steps	320,000	400,000	480,000	1.9 M
Batch size (in tokens)	524,000	524,000	524,000	262,000
Epochs	1	1.25	1.5	4
Total tokens	169 billion	211 billion	250 billion	515 billion
Total time (days)	1.8	6.9	7.2	30
Tokens/parameter	1,050	335	225	210
Max learning rate	1 × 10^−3^	6 × 10^−4^	4 × 10^−4^	2 × 10^−4^
Min learning rate	1 × 10^−4^	6 × 10^−5^	4 × 10^−5^	2 × 10^−5^
GPU count (A100)	8	8	16	16
MFU	43%	54%	53%	55%
Tokens/s	1,066,000	346,000	387,000	180,200
% memory footprint	43.75%	92.5%	95%	95%

All models used AdamW,^116^ with the following configuration: *ε* = $1\times 10^{-8}$, $\beta_{1}$ = 0.9, and $\beta_{2}$ = 0.95. We applied a weight decay factor of 0.1 and a gradient clipping threshold of 1.0 to regularize gradient values. Regarding optimizer scheduling, all models had 1,000 warm-up steps, where the learning rate was linearly increased up to the max learning rate. After that, during 90% of the training, we used a cosine learning rate decay from its maximum value to a minimum learning rate (10% of the maximum learning rate). For the last 10% of the training, the minimum learning rate is sustained as a constant. All models were trained using BF16 mixed precision, TF32 mode enabled for matrix multiplication operations, and FlashAttention 2, in addition to the Liger Triton kernels. Many of these configurations were estimated via experiments (i.e., short runs of ≈10,000 steps) or directly imported from the documentation of other LLMs of similar size.^14^^,^^22^^,^^92^^,^^115^^,^^117^^,^^118^

### Evaluation protocol

While training our base models, we saved several checkpoints for each model at intervals of approximately 10.5 billion tokens. For each checkpoint, we evaluated model performance using a small held-out dataset of 60,000 samples (randomly selected and excluded from GigaVerbo) to track perplexity as the training progressed. In addition, we implemented a comprehensive evaluation harness modeled after the work of Corrêa et al.,^19^ with additional benchmarks included. The benchmarks in our evaluation harness can generally be categorized into two types: native evaluations, specifically developed in Portuguese, and translated ones, consisting of English benchmarks machine translated into Portuguese. Table 5 lists all the evaluations used in our custom harness.

**Table 5:** Custom Portuguese evaluation harness

Benchmark	*n*-shot	Origin	Type	Metric
ENEM	3-shot	native	Q&A	acc
BLUEX	3-shot	native	Q&A	acc
OAB exams	3-shot	native	Q&A	acc
ASSIN2 RTE	15-shot	native	entailment	F1 macro
ASSIN2 STS	10-shot	native	similarity	Pearson
FAQUAD NLI	15-shot	native	entailment	F1 macro
HateBR	25-shot	native	classification	F1 macro
PT Hate Speech	25-shot	native	classification	F1 macro
TweetSentBR	25-shot	native	classification	F1 macro
CALAME-PT	0-shot	native	next word prediction	acc
ARC-Challenge	25-shot	translated	Q&A	acc norm
HellaSwag	10-shot	translated	Q&A	acc norm
TruthfulQA	0-shot	translated	Q&A	bleurt
LAMBADA	0-shot	translated	next word prediction	acc

Implementation settings for the evaluation harness used in our work are provided. To learn how to replicate our usage of this harness, please visit the evaluation section of our GitHub repository. Acc, acc norm, and bleurt stand for accuracy, accuracy normalized by byte length, and bilingual evaluation understudy with representations from transformers, respectively.

In total, this evaluation harness comprises 14 benchmarks, 10 of which are native to Portuguese, and four are machine translated from English datasets. The native benchmarks include ENEM,^119^ BLUEX,^120^ OAB,^121^ ASSIN2 RTE,^73^ ASSIN2 STS,^73^ FAQUAD NLI,^122^ HateBR,^74^ PT Hate Speech,^123^ and TweetSentBR,^75^ all integrated into the Brazilian Portuguese implementation of the Language Model Evaluation Harness,^124^ made available by Garcia.^125^ In the assessment of CALAME-PT, we had to create a custom evaluation protocol based on the work of Lopes et al.,^57^ i.e., all generations are performed deterministically without sampling in a zero-shot manner, with only exact matches being counted as a successful inference.

The remaining benchmarks, ARC-Challenge,^126^ HellaSwag,^127^ TruthfulQA,^128^ and LAMBADA-PT, are all evaluations tied to a machine-translated version of the original (English) datasets. ARC-Challenge, HellaSwag, and TruthfulQA are made available through a multilingual implementation of the Language Model Evaluation Harness by Lai et al.^76^ All few-shot settings for assessment remain the same as the one set for standard leaderboard comparisons (translations were generated via GPT-3.5-turbo). For LAMBADA-PT, a machine-translated version (via the Google Translate API) of the original LAMBADA test set,^129^ we employed the same evaluation protocol as CALAME-PT, given that both benchmarks involve the same primary task (i.e., zero-shot prediction of the final word in a given sentence).

Finally, to evaluate the "Instruct" versions of our base models, we developed a Portuguese chat evaluation dataset comprising 805 completion samples machine translated (via the Google Translate API) from the original Alpaca dataset.^56^ In this evaluation, our model’s outputs are compared to a reference standard (i.e., the original text-davinci-003 completions from the Alpaca dataset) and later judged by an automated annotator (GPT-4 Turbo) to determine their relevance, coherence, and adherence to the instruction prompts. In line with the evaluation methodology proposed by Dubois et al.,^130^ we use length-controlled win rates as our evaluation metric, given that these are highly correlated with human preferences and evaluations of pairwise comparisons.

### Alignment protocol

To offer a more easy-to-use version of our more capable models (i.e., 1b1 and 2b4), we implemented a fine-tuning process divided into two stages: supervised fine-tuning (SFT)^131^ and direct preference optimization (DPO).^132^

For the SFT step, we synthesized a small dataset containing over 600,000 samples of user-assistant interactions generated by other models that went through an alignment process. A description of this dataset can be found in Table 6. These fine-tuned models have special chat-delimiting tokens (i.e., <instruction> and </instruction>) added to their tokenizers, and training began from the final checkpoint of their respective base models (e.g., Tucano-1b1, step 480,000). Regarding hyper-settings, fine-tuning jobs performed another learning rate decay to 10% of the original minimal value achieved during training, with no warm-up steps and all other hyper-parameters unchanged. Each model was fine-tuned with a batch size of 262,000 tokens per optimizer step for four epochs.

**Table 6:** Composition of Tucano’s SFT dataset

Subset	Nº of Samples	%	Description
GPT4-500k-PTBR	565,536	83%	a machine-translated version of conversations with GPT-4
Orca-Math-PT	64,073	9.5%	a machine-translated version of Orca-Math dataset
Instruct-Aira v.3	50,000	7.5%	a collection of multi-turn conversations generated by user interactions with conversational LLMs

The various subsets used to create the SFT dataset for training the Tucano-Instruct models are outlined. The GPT4-500k-PTBR^86^ and Orca-Math-PT^133^ datasets do not specify the API or model used for translation. In contrast, the Google Translate API translated the Instruct-Aira v.3^134^ samples from English to Portuguese.

Finally, for the DPO step, we used the preference modeling dataset developed by Corrêa,^134^ which consists of 35,000 triplets comprising a user prompt, a preferred response, and a less preferred alternative. We design our DPO fine-tuning implementation on top of the Transformer Reinforcement Learning (TRL) library.^135^ We trained both models using their respective SFT versions as initial checkpoints. Regarding hyper-parameters, for both models, we used a cosine learning rate scheduler with a learning rate of $1\times 10^{-6}$ and a weight decay of 0.1. We set beta to 0.5, applied sigmoid as the loss function, and used zero label smoothing. We repeated the dataset for three epochs, with global batch sizes of 16 for the 1b1 model and 8 for the 2b4 model. This two-step alignment approach outputs our models’ Instruct versions: Tucano-1b1-Instruct and Tucano-2b4-Instruct.

## Results and discussion

This section presents and discusses results from our Portuguese evaluation harness, which was used to test the Tucano series and several other LLMs of comparable size. We will also present other pertinent metrics logged during the training and evaluation of our models.

### Learning curves for the Tucano series

**Figure 4:**
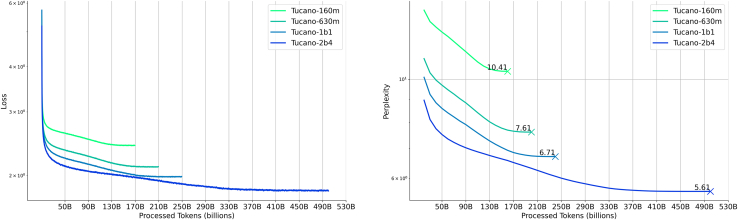
Learning curves for the Tucano series The left graph records the logged training loss for all 4 models. At the same time, the right one presents perplexity scores logged at intervals of approximately 10.5 billion tokens. To access the original logs of our training runs, visit our GitHub repository.

The Tucano series was pretrained on GigaVerbo, a large-scale Portuguese text dataset comprising over 145 M documents assembled and filtered from diverse, publicly available sources. To optimize training efficiency under data constraints, we followed the scaling heuristic proposed by Muennighoff et al.,^136^ which suggests that repeating data for 4–5 epochs yields marginal differences in loss compared to using unique data. Accordingly, for the smaller models in our series (160 M, 630 M, and 1.1 billion parameters), we selectively repeated high-quality subsets of GigaVerbo—as ranked by our learned filter—for up to four epochs (Table 7) while using four epochs of repetition across the entire filtered dataset for our largest model (2.4 billion parameters). Figure 4 shows the training loss and validation perplexity curves for all four base models. Consistent with scaling expectations, larger models achieved steeper reductions in loss and perplexity throughout training.

**Table 7:** GigaVerbo’s composition and filtering statistics

Subset	Original size	Filtered size	%	Repeat factor	Token count
monoHPLT-PT	58,244,012	37,291,607	64.03%	1	84,708,988,928
CrawlPT	43,846,974	29,427,715	67.11%	1	14,023,256,064
Multilingual-C4	16,092,571	13,849,412	87.10%	2	8,083,937,280
Common Crawl	12,470,998	10,527,584	84.42%	2	14,421,852,160
BlogSet-BR	4,321,181	2,411,590	55.81%	1	1,561,569,280
Instruct-PTBR	2,962,856	2,570,829	86.77%	4	1,141,768,192
Corpus Carolina	2,075,395	1,170,905	56.42%	1	1,018,951,680
UltrachatBR	1,255,091	1,2477,14	99.41%	4	1,652,916,224
Wikipedia	1,101,475	921,137	83.63%	4	551,403,520
CulturaX	999,994	883,550	88.36%	4	565,768,192
LegalPT	925,522	891,891	97.62%	4	1,313,269,760
Gpt4All	808,803	725,195	89.66%	4	381,650,944
Bactrian-X	66,994	55,685	83.012%	4	9,517,056
XL-SUM	64,577	64,467	99.83%	4	52,072,448
Dolly 15K	28,401	21,016	74.00%	2	3,698,688
CosmosQA	25,260	14,702	58.20%	1	2,074,624
ROOTS	10,740	5,448	50.72%	1	11,456,512
Total	145,300,844	102,080,447	70.25%	–	129 billion

An overview of the document counts in each subset of GigaVerbo, including their original size, the number of documents remaining after filtering, and the repetition factor used to create the training mixture for the Tucano models (160m, 630m, and 1b1), is provided. The token count column shows raw values before applying the repetition factor to the filtered dataset, which contains approximately 129 billion tokens, compared to 200 billion tokens in the unfiltered version. For training Tucano-2b4, our largest model, we repeated the filtered dataset for four epochs, resulting in a total training corpus of approximately 515 billion tokens.

### How does token ingestion correlate with benchmark performance?

As defined in our evaluation protocol, we saved a checkpoint for each model for every 10.5 billion tokens processed during training and ran our evaluation harness on it. This approach allowed us to systematically track and represent model performance as a function of time/token ingestion, which then enabled us to observe the relationship between model performance across several benchmarks and token ingestion on a plain causal language modeling regime without intentionally seeking to overfit a specific training (or evaluation) distribution.

If we assume that "the more a model is trained on new tokens, the more capable it becomes," as demonstrated by several works examining the scaling behavior of LLMs,^14^^,^^104^^,^^115^ we would expect to observe this phenomenon when evaluating our models with the custom evaluation harness we developed (which contains most of the evaluations documented by the studies reviewed in our literature analysis). To test this hypothesis, we calculated the Pearson product-moment correlation coefficients between our evaluation results and the number of tokens processed at each checkpoint. A positive correlation between token ingestion and benchmark performance would suggest a relationship between these variables, implying that performance improves as the model ingests more tokens. However, this anticipated pattern was only observed across a few benchmarks, as seen in Table 8.

**Table 8:** Correlation of benchmark results with token ingestion in the Tucano series

Benchmark	r_160m_	r_630m_	r_1b1_	r_2b4_
ENEM	0.10	−0.45	0.41	0.48
BLUEX	−0.06	0.21	0.52	0.00
OAB exams	0.34	0.21	0.28	0.24
ASSIN2 RTE	−0.30	**0.78**	**0.74**	0.34
ASSIN2 STS	0.58	0.22	**0.81**	−0.50
FAQUAD NLI	−0.31	0.00	0.00	0.17
HateBR	−0.56	**0.65**	0.48	−0.36
PT Hate Speech	−0.75	−0.15	0.31	0.49
TweetSentBR	0.17	0.50	0.33	**0.88**
**CALAME-PT**	**0.92**	**0.96**	**0.94**	**0.86**
**ARC-Challenge**	**0.76**	**0.69**	**0.82**	**0.61**
**HellaSwag**	**0.93**	**0.94**	**0.90**	**0.89**
TruthfulQA	−0.16	**0.66**	−0.05	−0.30
**LAMBADA**	**0.89**	**0.90**	**0.91**	**0.86**

All correlation scores for each benchmark against the different models are shown. The bolded scores correspond to a Pearson product-moment correlation value above 0.6, while the highlighted benchmarks are those where a positive correlation above 0.6 was found for all models, irrespective of size. To replicate these results, you can use the evaluation logs and code implementation available in our GitHub repository.

Even when considering the possibility that specific in-context learning abilities only emerge beyond certain model sizes, our results do not show a consistent trend across benchmarks of the same type, such as multiple-choice question-and-answer (Q&A) evaluations. For instance, benchmarks like ENEM and BLUEX show a moderate positive correlation only for the 1b1 model. Meanwhile, for the OAB exams (Brazilian Bar exam), performance appears entirely uncorrelated with the number of tokens processed, despite over 4 billion tokens from our dataset originating from the legal domain, regardless of model size. We initially hypothesized that model performance might only exceed random chance for benchmarks like ENEM, BLUEX, and OAB exams once the models surpass a certain parameter threshold (e.g., 7 billion), which would explain the poor performance of smaller models. However, this does not explain why other similarly structured benchmarks, such as ARC-Challenge and HellaSwag, show clear scaling effects even in smaller models.

At the same time, for all sub-billion parameter models, we find instances where "training hinders benchmark performance," i.e., inverse scaling. This is especially true for our 160-M-parameter model, where, for several benchmarks, its performance worsens as the model advances its training. Also, for evaluations like HateBR and ASSIN2 STS, we again see this phenomenon afflicting our 2b4 model, where training causes the models to become worse than a random guesser. At the same time, performance on benchmarks like the Portuguese native FAQUAD NLI seems completely uncorrelated with token ingestion.

These findings challenge the effectiveness of current benchmarks in accurately assessing the quality of large-scale pretraining runs—especially when training is primarily based on noisy, web-crawled data. More specifically, we believe these benchmarks may require a level of specialization that our native Portuguese datasets inherently lack, potentially necessitating synthetic augmentation to improve performance in these evaluations. This hypothesis is backed by the fact that language modeling capabilities did not directly translate into success on these benchmarks, regardless of the number of training tokens and with the model size ranging from 160 M to 2.4 billion parameters. Moreover, we hypothesize that achieving strong performance on such evaluations (i.e., surpassing random-guessing baselines) could be attributed to either overfitting to the benchmark’s format—such as multiple-choice Q&A in OAB exams or ENEM tests—or simple chance. However, due to the lack of transparency in many prior works regarding critical details such as pretraining and fine-tuning datasets, it remains difficult to empirically explain the reported performance^38^^,^^59^^,^^70^ and requires further investigation.

**Figure 5:**
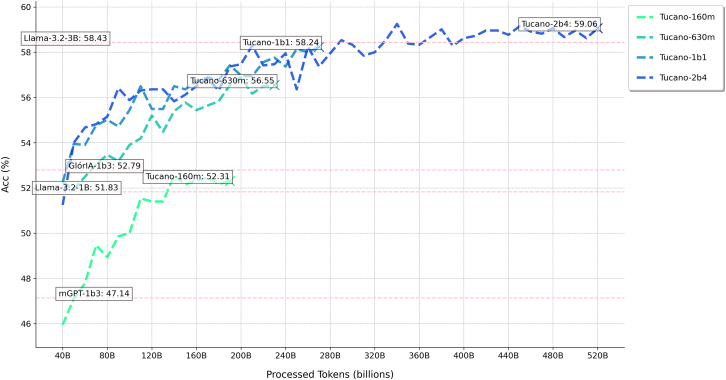
Benchmark score progression for CALAME-PT across the Tucano series The table presents the evaluation progression of the Tucano series on the CALAME-PT benchmark.^57^ It also compares benchmarking results for other models of similar size, including Glória-1b3, mGPT-1b3, and Llama-3.2-3b, highlighting Tucano-2b4’s superior performance.

Despite these inconsistencies, we identified several benchmarks where increased pretraining led to (>60%) positive correlation with performance across the entire model series. Benchmarks such as CALAME-PT (Figure 5), LAMBADA, HellaSwag, and the ARC-Challenge consistently showed improvement as causal language modeling pretraining scales. These benchmarks, therefore, seem to serve as reliable indicators of model performance and capabilities when training native Portuguese LLMs with plain Common Crawl data. This is not to say that the other benchmarks are not helpful but rather that they require a domain specialization that goes beyond what GigaVerbo possesses, suggesting paths for future dataset augmentation. These insights could assist other practitioners in determining areas of improvement for which one could contribute to creating future, more augmented pretraining corpora.

### Benchmarking comparisons for base models

Focusing only on the benchmarks that showed a significant correlation between language modeling pretraining and performance, we obtained the results shown in Table 9. According to our evaluation protocol, our largest models outperformed several multilingual and natively pretrained LLMs across nearly all benchmarks, including the (at the time) recently released Llama-3.2-1b, which was trained on a far larger dataset than GigaVerbo. Our models also outperformed larger multilingual models, such as Bloom-1b7, in benchmarks like CALAME-PT and LAMBADA. Considering all benchmarks in our evaluation suite, our series outperforms all models listed in Table 9 except those from the Llama-3.2 series. A more complete table of evaluation results can be found in our GitHub.

**Table 9:** Evaluation comparisons for CALAME-PT, LAMBADA, ARC-Challenge, and HellaSwag

Model	Average	CALAME-PT	LAMBADA	ARC-CHALLENGE	HellaSwag
Llama-3.2-3B	52.00	58.43	49.1	43.25	57.2
**Tucano-2b4**	43.58	59.06	37.67	30.43	47.17
Llama-3.2-1B	42.95	51.83	41.02	33.5	45.44
**Tucano-1b1**	41.55	58.24	34.7	30.43	42.84
Gemma-2b	40.38	51.16	39.88	37.95	32.53
Bloom-1b7	40.37	55.64	31.98	30.34	43.52
**Tucano-630m**	39.5	56.55	33.13	28.89	39.41
Gemma-2-2b	39.21	56.7	47.1	24.19	28.85
Bloom-1b1	38.18	52.94	30.22	29.83	39.74
GlórIA-1b3	36.05	52.79	27.71	26.67	37.04
**Tucano-160m**	35.14	52.31	28.16	27.01	33.07
XGLM-564m	34.55	50.58	27.42	25.56	34.64
Bloom-560m	34.32	49.95	25.44	24.74	37.15
TTL-460m	33.78	49.42	23.29	29.4	33.00
mGPT-1b3	31.81	47.14	29.92	23.81	26.37
TTL-160m	30.78	46.72	20.98	26.15	29.29
Lola-v1	30.19	26.4	18.32	30.42	45.61
GPorTuguese-2	28.92	40.61	22.98	22.48	29.62

The evaluation benchmark scores for our models compared with models of similar size are shown. All evaluations for all benchmarks that form our custom Portuguese harness are available on our GitHub repository.

### Benchmarking comparisons for assistant models

As shown by other studies where small-scale assistant models of comparable size^14^^,^^137^ were trained, we also found that the fine-tuning/alignment process usually degrades the performance of the foundational model on specific benchmarks. For instance, we observed that while alignment improved the controllability and usability of our models, it also reduced performance on particular benchmarks. However, when evaluated on our custom AlpacaEval benchmark, a more appropriate benchmark for evaluating assistant models, our Instruct models gave promising results (Table 10). More specifically, the Tucano-Instruct models outperform much larger models (e.g., Sabiá-7b and Gervásio-7b) and approximate models like the ones from the Llama-3.2 series.

**Table 10:** Evaluation comparisons for Alpaca-Eval-PT

Model	Avg. length	Wins	Base wins	LC win rate (%)	SE
Llama-3.2-3B-Instruct	1,609	257	548	21.06	0.075
**Tucano-2b4-Instruct**	1,843	151	654	13.00	0.071
**Tucano-1b1-Instruct**	1,667	124	681	8.80	0.083
Llama-3.2-1B-Instruct	1,429	99	706	7.15	0.057
TTL-460m-Chat	1,333	28	777	2.84	0.059
Sabiá-7b	5,011	1	804	0.076	0.0043
Gervásio-7b (PT-BR)	5,740	1	804	0.026	0.0016

The evaluation benchmark scores for our assistant models when evaluated on 805 prompts from the Alpaca-Eval-PT benchmark are shown. Length-controlled win rates and standard errors for the different models we were able to evaluate are also presented. All evaluations from this custom benchmark harness are available on our GitHub repository, where the reader can also find resources to replicate our results.

### Energy consumption and carbon emissions

Following the example of past works^138^^,^^139^^,^^140^^,^^141^ and acknowledging the environmental costs of large-scale deep learning,^142^^,^^143^^,^^144^^,^^145^ we tracked our energy use during training, experiments, and evaluation. We measured the energy consumption and estimated carbon emissions for every checkpoint created during our training runs and experiments. All estimations were made using the 2023 estimations of the carbon intensity of Germany’s energy grid (0.37 KgCO_2_ eq/KWh), which, according to Lottick et al.’s^146^ methodology, can be used to infer carbon emissions (CO_2_ eq) by multiplying the carbon intensity of the energy grid by the total energy consumption of a given experiment. Table 11 summarizes the energy and carbon footprint related to our work.

**Table 11:** Energy consumption, training duration, and carbon emissions of model development

Model	Duration (h)	Training (kWh)	Exp. (kWh)	Emissions (KgCO_2_eq)
160m	44	235	200	160
630m	170	920	125	387
1b1	194	2,600	335	1,085
2b4	860	11,860	400	4,536
Total	1,268	15,615	1,060	6,168 KgCO_2_eq

For each model, the duration of its training run, the energy consumption related to that run, the energy consumption regarding experimentation and evaluations, and the total estimated carbon emissions regarding the development of that model size are shown. The training of the instruct versions is also accounted for in each respective model. To minimize energy consumption, we performed almost all of our experiments using the smaller version of our models. According to our logs, we utilized around 5,900 GPU h across training, translating to an estimated cost of approximately 5,990 USD, assuming a rate of 1.1 USD per h per A100 GPU. From the total of 16,675 kWh used, a significant portion (≈6%) was used to run experiments and evaluations, totaling 6.1 tCO_2_eq in emissions.

Deep learning research is fundamentally driven by experimentation and heuristic approaches. Although many studies attempt to document training procedures,^103^^,^^115^^,^^117^ offering valuable guidelines for configuring models and their training environments, these published (or documented) procedures rarely provide universal solutions. Hence, the heuristic challenges and the current deficiencies in training documentation force researchers to expend resources and energy that could have been avoided when developing new models. Meanwhile, several factors shape the carbon footprint of deep learning, including the unique characteristics of each experiment and the infrastructure supporting it. In our experience, we frequently needed to fine-tune hyper-parameters, adjust preprocessing strategies, and conduct exploratory experiments to achieve good results. However, this reliance on experimentation has significant environmental implications. To address this issue, we performed most experiments using our smaller models, as experimenting with the larger models (e.g., 2b4) would have led to a much higher increase in CO_2_ emissions, which we aimed to avoid. In short, LLM development is computationally demanding, with a substantial portion of energy consumption occurring outside the training runs.

### Future works

The Tucano series significantly contributes to the Portuguese NLP community in several ways. First, all models are open source, reproducible, and trained on the largest monolingual Portuguese dataset to date. To the best of our knowledge, the scale of monolingual Portuguese pretraining in this study is unprecedented in the literature. All models, along with intermediary checkpoints, datasets, code implementations, and logs, are freely accessible through the repositories associated with this study. Table 12 summarizes the availability of the artifacts mentioned in our literature review compared to our work. In a follow-up study, we intend to do the following:

(1) Expand GigaVerbo with more high-quality Portuguese text. Future studies should seek to enrich our pretraining corpus with more high-quality tokens, like academic papers, books, and other forms of high-quality text. Ambitiously, we should aim to reach the trillion-token range. At the same time, it would be interesting to conduct ablation studies on GigaVerbo to determine the impact of different dataset components and identify which subsets contribute most effectively to model performance.
(2) Augment GigaVerbo with synthetic data. While this approach was not explored in our current study, synthetic data augmentation has been proven in other works to bolster model performance in many specific domains (e.g., coding and storytelling).^92^ In the future, augmenting GigaVerbo with this type of data could improve its representative power in domains where, in its current state, it is found to be lacking.
(3) Explore downstream uses of Tucano models: future studies can use the models from the Tucano series as foundations for future developments, like multimodal Portuguese LLaVas,^147^ Portuguese embedding models,^148^ or more capable filters and guardrails.
(4) Increase model scale to larger architectures, such as 3, 7, and 13 billion parameters. Scaling up to larger model sizes would enable us to better understand how benchmark performance changes with model size and to determine whether specific benchmarks correlate more strongly with language modeling pretraining only when models exceed a certain size threshold.
(5) Develop new and more comprehensive benchmarks for Portuguese. Our results indicate that Portuguese evaluation benchmarks for generative language models require improvement. Future research to advance Portuguese NLP should focus on either developing more effective benchmarks or refining existing ones to better capture the impact of pretraining and provide a more precise correlation between pretraining depth and performance across various language tasks.

**Table 12:** Open-source availability and reproducibility of Portuguese language models

	Model	Data	Code	Logs	#models	#ckpts
Tucano	✔	✔	✔	✔	6	111
TeenyTinyLlama	✔	✔	✔	✔	3	70
GPorTuguese-2	✔	✔	✔	✔	1	1
PTT5	✔	✔	✔	✘	6	1
RoBERTaLexPT	✔	✔	✘	✘	2	3
Albertina	✔	✔	✘	✘	8	1
BERTimbau	✔	✔	✘	✘	2	1
DeBERTinha	✔	✔	✘	✘	1	1
Gervásio	✔	✔	✘	✘	2	1
PTT5-v2	✔	✘	✘	✘	4	1
BERTabaporu	✔	✘	✘	✘	2	1
Glória	✔	✘	✘	✘	1	1
Sabiá	✔	✘	✘	✘	1	1
Sabiá-2	✘	✘	✘	✘	2	none
Sabiá-3	✘	✘	✘	✘	1	none

Portuguese language models regarding the open-source availability of models, datasets, code, logs, the total number of models (#models), and checkpoints (#ckpts) are compared. In terms of open (and reproducible) development, many aspects of past studies are indeed closed. Save for rare exceptions,^19^^,^^28^^,^^29^many studies only make available "end products" devoid of logs, datasets, or code implementations, making the reproduction of LLM development a task that requires constant rediscovering. Given the level of computing needed to practice deep learning at such scales, a lack of reusable code and materials can seriously slow down the Portuguese NLP community’s progress while hindering its sustainability.

### Conclusion

In this study, we introduced the Tucano series, a collection of open-source LLMs designed to advance NLP for Portuguese. Our work covered the entire development pipeline, from dataset creation and filtration to hyper-parameter tuning and evaluation, emphasizing openness and reproducibility. These efforts contribute capable models, large datasets, and tools to the Portuguese NLP community to set a standard for transparent and replicable research practices. Moreover, our critical assessment of the field highlighted ongoing challenges, particularly around evaluation methodologies and result interpretability, which will only be solved if the community shifts toward a more rigorous and reproducible developmental framework. Ultimately, we hope the work initiated here will be extended to other low-resource languages, fostering a more equitable and sustainable NLP ecosystem globally.

## Resource availability

### Lead contact

The lead contact is Nicholas Kluge Corrêa. He is a postdoctoral researcher at the Center for Science and Thought at the University of Bonn (Bonn, NRW, Germany). His contact email is kluge@uni-bonn.de.

### Materials availability

This study did not generate new materials.

### Data and code availability

Our source code is available at GitHub (https://github.com/Nkluge-correa/Tucano) and has been archived at Zenodo.^149^ Our dataset is available and has been archived at Hugging Face^150^ (https://huggingface.co/datasets/TucanoBR/GigaVerbo).

## Acknowledgments

The authors gratefully acknowledge the granted access to the Marvin cluster hosted by the 10.13039/501100008131University of Bonn along with the support provided by its High Performance Computing & Analytics Lab. The authors would also like to acknowledge their own personal funding agencies. N.K.C. is funded by the Ministerium für Wirtschaft, Industrie, Klimaschutz und Energie des Landes Nordrhein-Westfalen (Ministry for Economic Affairs, Industry, Climate Action and Energy of the State of North Rhine- Westphalia) as part of the KI.NRW-flagship project "Zertifizierte KI" (Certified AI). A.S. is funded by the 10.13039/501100001659Deutsche Forschungsgemeinschaft (DFG, German Research Foundation) as part of the CRC 1639 NuMeriQS – project no. 511713970.

## Author contributions

N.K.C. contributed to the project’s idealization, the software stack’s implementation, dataset creation, training, and evaluation of the models, as well as writing the article and documenting the repositories. A.S. contributed to the optimization of the software stack, training, and evaluation of the models, as well as the article’s writing. S. Falk contributed to implementing the carbon tracking methodology, monitoring training runs, and writing the article. S. Fatimah contributed to developing the datasets, including deduplication and cleaning, writing the article, and documenting the repositories.

## Declaration of interests

The authors declare no competing interests.
